# Humanized Mice for Studies of HIV-1 Persistence and Elimination

**DOI:** 10.3390/pathogens12070879

**Published:** 2023-06-27

**Authors:** Chen Zhang, Lubaba A. Zaman, Larisa Y. Poluektova, Santhi Gorantla, Howard E. Gendelman, Prasanta K. Dash

**Affiliations:** Department of Pharmacology and Experimental Neuroscience, College of Medicine, University of Nebraska Medical Center, Omaha, NE 68198, USAsgorantla@unmc.edu (S.G.);

**Keywords:** HIV-1, latency, experimental cell systems, animal models, humanized mice

## Abstract

A major roadblock to achieving a cure for human immunodeficiency virus type one (HIV-1) is the persistence of latent viral infections in the cells and tissue compartments of an infected human host. Latent HIV-1 proviral DNA persists in resting memory CD4+ T cells and mononuclear phagocytes (MPs; macrophages, microglia, and dendritic cells). Tissue viral reservoirs of both cell types reside in the gut, lymph nodes, bone marrow, spleen, liver, kidney, skin, adipose tissue, reproductive organs, and brain. However, despite the identification of virus-susceptible cells, several limitations persist in identifying broad latent reservoirs in infected persons. The major limitations include their relatively low abundance, the precise identification of latently infected cells, and the lack of biomarkers for identifying latent cells. While primary MP and CD4+ T cells and transformed cell lines are used to interrogate mechanisms of HIV-1 persistence, they often fail to accurately reflect the host cells and tissue environments that carry latent infections. Given the host specificity of HIV-1, there are few animal models that replicate the natural course of viral infection with any precision. These needs underlie the importance of humanized mouse models as both valuable and cost-effective tools for studying viral latency and subsequently identifying means of eliminating it. In this review, we discuss the advantages and limitations of humanized mice for studies of viral persistence and latency with an eye toward using these models to test antiretroviral and excision therapeutics. The goals of this research are to use the models to address how and under which circumstances HIV-1 latency can be detected and eliminated. Targeting latent reservoirs for an ultimate HIV-1 cure is the task at hand.

## 1. Introduction

Despite advances in antiretroviral therapy (ART) toward improving the quality and longevity of life for people infected with human immunodeficiency virus (HIV) (PLWH), the medicines must be taken for life. Adherence is fraught with personal, social, behavioral, drug resistance, and pharmacologic limitations [1,2]. While ART can prevent viral transmission and suppress viral transmission [1,3], it is not a cure. The virus persists as a latent infection in multiple cell and tissue compartments. This includes populations of resting memory CD4+ T and myeloid cells [4]. Indeed, immediately after infection, a stable integration of the viral genome into the genomic DNA of the host cell occurs with limited changes in viral gene expression [5]. A reservoir of latent virus is quickly established. This occurs within a few days after viral infection and even before the virus is detectable in systemic circulation [6,7]. Current ART regimens cannot affect latent viral infection, resulting in viral rebound once therapy is discontinued. While early ART administration can lower the number of infected cells via viral suppression and limiting spread, it cannot eliminate the persistent, latent HIV-1 proviral DNA. Studies of HIV latency necessitate a multifaceted approach, integrating the disciplines of virology, immunology, and cell biology. Studies of HIV latency are of immediate importance as viral elimination cannot be achieved without a thorough understanding of the mechanisms of viral persistence. This enables researchers to devise new methods for eliminating the virus from its infected human host.

## 2. HIV Reservoirs

Despite the significant advances made in understanding HIV reservoirs, there are still differences of opinion on how to best to define them, which will impact strategies aimed at eliminating infectious reservoirs. The “latent HIV reservoir” is where persistent, replication-competent viral populations are present during and following ART [8]. A latent reservoir consists of cells that maintain an HIV infection in a dormant state for an extended period following ART. Recent evidence suggests that viral clones represent a dynamic and actively dividing cell population that occurs through homeostatic proliferation or in response to cognate antigen stimulation [9]. In both circumstances, virus production occurs in a sub-population of the infected cell clones while in others, HIV remains latent despite continuous cell division. Therefore, any type of infected cell that can sustain the presence of a virus in a dormant state and periodically divide without activating the latent virus would fit the definition of a latent cell reservoir. CD4+ memory T cells fit this description and have been the most extensively studied [10]. However, other cell types that include mononuclear phagocytes (MP; monocytes, macrophages, dendritic cells, and microglia) as well as endothelial cells and astrocytes in the central nervous system (CNS) also act potential reservoirs of infection [11,12,13]. CD4+ T cells are the widely studied signature for virus persistence and latent infection with the ability to sustain viral infection with a half-life of up to 44 months [3,14]. The stability of these cells in acting as latent viral reservoirs is well known [15]. The decay of memory T-cells ranges from 8 to 15 years, which explains how these cells, when infected with virus, can sustain immunologic memory against the pathogen for the entirety of a patient’s life. This can also explain how the virus can be sustained for a long period of time in the face of an active, competent immune system [16]. Moreover, each HIV-1 integration event is unique; cells may contain the same integration site, indicating that the "clonal expansion" and multiplication of infected cells occurs [17,18]. Oligoclonal free virus, a byproduct of clonally expanded cells, was found in trace amounts in the plasma of ART-suppressed patients [17,19], implying their role in the production of residual viremia in some patients and posing a greater challenge in achieving an HIV-1 cure [20,21,22]. These cellular reservoirs exist within tissue reservoirs that include the gut mucosa, lymph nodes, bone marrow, spleen, lung, liver, kidney, skin, adipose tissue, reproductive organs, and brain, demonstrating that each site harbors latent infected cells [23,24,25,26,27,28,29,30].

## 3. Pitfalls for Studies of Latent HIV Infection 

Studies on HIV-1 latency are hindered by the difficulty of performing in vivo studies as the cells that carry proviral DNA are low in abundance. The resting CD4+ T cell reservoir with integrated provirus accounts for less than 0.05% of the entire resting cell population. This suggests that productively infected cells have higher rates of turnover. This may be partly due to apoptotic and pyroptic death caused by direct viral cytopathicity. Alternatively, host cytotoxic T-lymphocytes (CTLs) eliminate productively infected cells. Hence, most of the cells containing integrated provirus rarely persist to switch into a memory state [31]. It is also difficult to distinguish latently infected from uninfected cells without activation [32]. There is a lack of definitive biomarkers that differentiate latently infected from uninfected resting CD4+ T cells. 

## 4. Model Systems for Studies of HIV Reservoirs

### 4.1. Cell-Based Model Systems 

The selection of an appropriate model system is necessary when studying HIV latency and reactivation. Given the human host specificity of HIV-1, there are few animal models that can replicate long-term HIV infection and latency [33]. Consequently, in vitro models with primary and transformed cell lines are used to address the multifaceted challenges of virus–cell interactions. The HIV latencies observed in patients are affected by cell–tissue locations and environment and remain poorly understood [33]. Primary CD4+ T and myeloid cells recovered from peripheral blood mononuclear cells or lymphoid tissues [34,35] are the most commonly studied cell types when addressing latency [36]. A primary CD4+ T cell model was developed from PLWH memory CD4+ T cells that was subjected to two rounds of activation. Despite having similar reactivation patterns to latency-reversing agents (LRAs), CD4+ T cells can only survive for up to eight weeks [37]. Several groups have also attempted to mimic infection in activated CD4+ T cells by collecting and isolating CD4+ T cells from people without HIV (PWoH) and activating the cells [38,39]. The first reported model isolated CD4+ T cells from PWoH with combinations of a CD3 antibody and interleukin-2 (IL-2). The cells were then infected with replication-competent virus, and the infection progressed to quiescence (latency) [38]. Another study employed similar methods for the activation of CD4+ T cells, followed by their infection with HIV-1NL4-3 and HIV-1 NLENG1-IRES [39]. The system allowed the latent infection to be studied for up to two months. These approaches have also been used to investigate HIV vectors lacking multiple HIV genes—tat, env, gag, and vif—that are needed to infect activated cells [40,41]. An ex vivo primary cell system can mimic in vivo latency. Cells were transduced using a B cell lymphoma gene (*Bcl2)* to increase their lifespan [42]. Other studies demonstrated that ex vivo CD4+ T cells obtained from PLWH could be reactivated by various classes of LRAs; however, CD4+ T cells infected in vitro could be activated by protein kinase C (PKC) agonists [35]. HIV-1-specific CD8+ T cells fail to eliminate CD4+ T cells; however, they are able to eliminate CD4+ T cells infected with outgrown virus from the same individual [43]. Cell procurement from PLWH poses challenges [44] as the longevity of primary cells in culture is limited [42] and another hurdle is the necessity if manipulation of these replicate cells using transfection or electroporation to introduce the exogenous genes, proteins, or HIV-specific therapeutic agents into them. Primary cells can contain non-replication-competent viruses and have different integration sites [45] that could affect active viral transcription and large numbers of biological replicates are required to perform mechanistic studies and to reach statistical significance [33].

To generate meaningful data sets, researchers have employed immortalized cell lines that include CD4+ T cells (Jurkat, CEM, MOLT4, THP1, and SupT1) and the promonocytic U1 cell lines to study viral latency [46,47]. The advantage of using in vitro model systems is that large numbers of infected cells are used to evaluate gene expression and cell phenotype. Notably, the levels of involvement of transcription machinery in studying latency, specifically positive-transcription-elongation-factor-b-mediated pathways, are significantly elevated in the tested cell lines when compared to the primary resting CD4+ T cells and macrophages. Components of cellular transcription machinery are unique between cells, which can result in different experimental results when studies are performed to evaluate multiple stages of HIV transcription [48,49]. Also, resting primary CD4+ T cells carry latent HIV-1 in the G0 stage of the cell cycle [50,51,52]. Taking this into consideration, a major drawback of using transformed cell lines to study viral latency is that they may not accurately reflect what occurs in CD4+ T cells that carry latent HIV-1 in vivo. 

Yet another complexity in assessing the latent viral reservoir are the inherent differences between CD4+ T and myeloid cells in their carrying capacities and transcriptional machineries that support infections. Both cell types are known reservoirs of HIV. Indeed, tissue macrophages and microglia, along with circulating monocytes, can play roles in bearing latent HIV. These cells can have longer lifespans and resist virus-induced cytopathology [53] and are resistant to CTL cell elimination [54]. Additionally, myeloid cell reservoirs in the brain and lymphoid organs may show differential responses to antiretroviral drugs [55,56]. The previous notion that macrophages lack the capacity for self-renewal has been challenged by recent findings that demonstrate a more prominent role of macrophages as HIV reservoirs [57,58,59]. Additionally, the lack of immune-mediated viral clearance of infected macrophages may make the replication-competent carriage of latent virus and ultimate viral rebound in these cells more likely during therapy interruption [58,59,60]. Additional studies on viral activation in tissue macrophages that include the broader use of newly minted viral outgrowth assays are needed [61]. This need is supported by recent studies that show monocyte-derived macrophages (MDMs) as sources of rebound virus [62,63,64]. Future studies must account for the tissue and cellular environments. This can affect, for example, macrophage polarization and ultimately, HIV reactivation [61]. Moreover, LRAs known to be effective in reversing HIV latency in CD4+ T cells have different effects in macrophages. These factors all require further attention. 

Astrocytes, which represent some of the most abundant cell types in the brain, can harbor HIV [65,66]. The cells are involved in the secretion cellular and viral neurotoxic factors, including Tat [67]. They may affect the clinical signs and symptoms of HIV-associated neurocognitive disorders (HANDs) [68,69,70,71]. Despite prior studies demonstrating HIV infection in astrocytes [72,73,74], their role as viral reservoirs remains incompletely defined [75,76,77,78,79]. The presence of HIV-1p24 in astrocytes remains uncertain beyond 60 days after exposure to the virus [80,81]. Studies of HIV-1 infection in astrocytes were performed in cells with differential outcomes following viral exposures [82,83], or in cells isolated and differentiated from human fetal tissues [84]. The findings have not reliably allowed such virus-exposed cells to be used as accurate platforms for studies of HIV-1 latency. Newer approaches to studying viral latency in astrocytic cells have become available through HNSC-100, a human neural stem cell line. These cells may mimic the non-dividing nature of mature astrocytes in the adult human brain and are being used to analyze LRAs and inhibitors of latency reactivators [85]. However, suitable cell models remain in demand for studying HIV latency in CNS tissue compartments. 

### 4.2. Nonhuman Primates (NHPs) 

Over the last two decades, SIV/SHIV-infected NHP models of HIV-1 have been instrumental in studies of viral latency and cure research. This is based on the similarity at the cellular and molecular levels of the infection life cycle, disease progression, and immune responses observed [25,86,87,88]. In addition, when using NHPs, researchers can employ analytical treatment interruption to address questions relevant to HIV latency, persistent viral reservoirs, and cures which are risky and unethical to conduct in PLWH [89,90,91,92]. 

NHP models have allowed for the evaluation of novel therapeutic strategies that include immune regulators and boosters [93,94,95,96], LRAs [92,97,98], broad neutralizing antibodies (bnAbs) [89,90,99,100], HIV-specific chimeric antigen receptor T cell (CAR T) therapy [91,101,101,102], and CRISPR-based gene editing [103]. Prior studies tested antiviral efficacy against SIV/SHIV infection by measuring viral load, host antiretroviral immunity, and disease protection. However, few studies achieved control of SIV/SHIV latency [89,99,103,104]. Prior works have, nonetheless, demonstrated undetectable plasma SHIV after the administration of the bnAb PGT121. Here, a significant reduction in proviral DNA was demonstrated in the peripheral blood, gastrointestinal mucosa, and lymph nodes within weeks of treatment [104]. A delay in viral rebound after the antibody infusion was observed. Although the bnAb was not able to eliminate the virus from latent reservoir sites, it was able to reduce the reservoir size [104]. Similarly, in SHIV-infected macaques treated with five bnAbs, strong viral suppression was observed with a delay in viral rebound in less than five weeks compared with viral rebound seven days after single-bnAb infusions. The data indicate that combinations of bnAbs showed clearer advantages over single-bnAb therapy [99]. Regarding viral elimination studies in macaques, thus far, a single study reported a functional cure in a subset of macaques receiving a combination of a TLR agonist and antibody therapy [89]. Five out of eleven macaques in a dual-treated group had no viral rebound at 200 days after ART discontinuation, while the virus rebounded in most (10/11 and 9/11) macaques belonging to the single-treatment groups. AAV-mediated CRISPR-Cas9 targeting SIV LTR-Gag with daily ART in macaques was used to excise the latent provirus [103]. The authors reported an excision of SIV proviral DNA from the circulatory lymphocytes, spleen, lung, and lymph nodes of the treated animals at 6.5 months post infection, with differential efficiency among the animals. Interestingly, a substantial reduction (38–95%) in viral DNA was detected in the lymph nodes of all treated animals, suggesting the potential efficacy of CRISPR-mediated treatments. 

Though NHP models have contributed to the characterization of latent reservoirs and the discovery/evaluation of novel therapeutic strategies, the dissimilarities in the structural and virological signatures of SIV and HIV-1 are major limitations to using SIV-infected macaque models in a translational approach [86,105]. For this reason, SIV-infected NHPs are presumed to respond differently to antiretroviral agents such as different classes of ART regimens, LRAs, and HIV-1-envelope-specific bnAbs. Though chimeric SHIV overcomes this barrier with the insertion of the HIV-1 envelope gene [106,107], several researchers have argued that the SHIV lacks naturally occurring broad viral diversity [108] and is thus less reflective of natural reservoir establishment in which a versatile founder virus is engaged. In fact, recent work revealed the existence of altered, persistent viral reservoirs between SIV, SHIV, and HIV1/2 infections following ART-mediated viral suppression. These results highlight the mechanistic complexity of strain-specific viral treatment responses [109]. 

In addition, the higher cost of animal maintenance, longer study period, and high laboratory and personnel requirements compared to rodent models are other practical limitations for latency and cure studies using NHP models. In summary, NHP viral latency studies have uncovered novel therapeutic pathways for viral suppression. However, there remains an immediate need for alternatives. These include small animal model systems that can accurately mimic natural HIV infection and disease progression while providing insights into established viral reservoirs. Such models can be employed to facilitate targeted therapeutic studies designed to improve viral suppression and elimination.

## 5. Rodent Animal Models 

Rodents are the preferred small animal model systems for use in infectious disease research [110,111] because they mirror the human system in many ways and have well-characterized immune systems. However, due to the human host specificity of the HIV virus, it is very difficult to find an ideal mouse model for studying HIV latent reservoirs and the cure strategies targeting them. One earlier model, the EcoHIV infection model, in which parts of HIV-1 gp120 were replaced with gp80 from the ecotropic murine leukemia virus, allowed for permissive HIV infection in conventional mice [112]. Recent studies by different research groups have shown neurocognitive impairment in an EcoHIV model without any significant CD4+ cell depletion [113]. This model is also being used by researchers to investigate the roles of infected macrophages and monocytes to understand how the persistence of HIV affects cognitive function despite ART, using molecular and behavior tests [114]. However, the lack of a proper HIV disease progression pattern is a major limitation of this model for latency and cure studies. In addition, the considerable differences between murine and human immune systems in terms of immune cell profiles, innate and adaptive immune responses, and the production of HIV-1-associated antiviral cytokines make this model less ideal for studying long-term HIV latency and assessing therapeutic efficacy [115]. Thus, humanized mice that can recapitulate a functional human immune system and mimic a natural HIV infection process remain preferred for HIV persistence studies [116]. 

Currently, several humanized mouse models are being used by researchers for HIV-related studies and are mostly developed from severe immunodeficient backgrounds in which the mice are engrafted with human immune cells or tissues. The journey of the development and generation of humanized mice began with the discovery of an autosomal recessive mutation in a DNA-dependent protein kinase, Prkdc*scid* (severe combined immunodeficiency, SCID), on the CB17 mouse strain background [117]. SCID mice have severely impaired lymphopoiesis and differentiation; thus, they lack their own functional T and B lymphocytes [117]. In 1988, McCune et al reported the co-transplantation of human fetal liver hematopoietic cells (fetal thymus and lymph nodes) into SCID mice for the first time. Though this pioneering study showed reconstituted mature human T and B cells in mouse peripheral circulation 6–7 weeks post transplantation [118], the human T cell population and human IgG were found to be transiently present because of graft-versus-host disease (GVHD), and the model lacked a sustained hematopoietic supportive environment [118,119]. Shortly thereafter, Namikawa et al. found that the co-transplantation of human fetal thymus and intact fetal liver fragments into SCID mice could support human hematopoiesis for up to 11 months, marking their model as the first humanized mouse model for HIV studies [120]. 

In addition to SCID, further mutations targeting the common cytokine receptor gamma chain (*γ*c or CD132) resulted in the generation of more immunodeficient mice strains: the NOD.Cg-*Prkdc^scid^ Il2rg^tm1Wjl^* (NSG), NODShi.Cg*Prkdc^scid^ Il2rg^tm1Sug^* (NOG), and BALB/c-*Rag2^null^ IL2rg^null^* mice (BRG) strains [121,122,123,124], which took humanized mouse research progress one step forward. Since the common cytokine receptor gamma chain is shared by a family of cytokines, including IL-2, 4, 7, 9, 15, and 21, and is crucial for the development and survival of NK cells and lymphocytes, the knockout or truncated version of *γ*c further reduced the risks of GVHD generation and enhanced the survival of the human cell engraftment in mice [125]. More advances have been made recently by knocking-in cytokine-encoding genes, for example, M-CSFh/h IL-3/GM-CSFh/h SIRPah/h TPOh/h RAG2−/− IL2Rg−/−, which led to support for the establishment of human cells in (MISTRG) mice [126]. Another mouse model with chimeric human–mouse class II transgenes, the NOD.Cg-*Rag1tm1Mom Il2rgtm1Wjl* Tg (DRAG) mouse, allowed for the engraftment of enhanced HLA-DR-matched hematopoietic stem cells (HSCs), which subsequently supported better human T and B cell development [127]. These most recent advances made in humanized mouse models further boost the planning and execution of infectious disease studies. 

**Figure 1:**
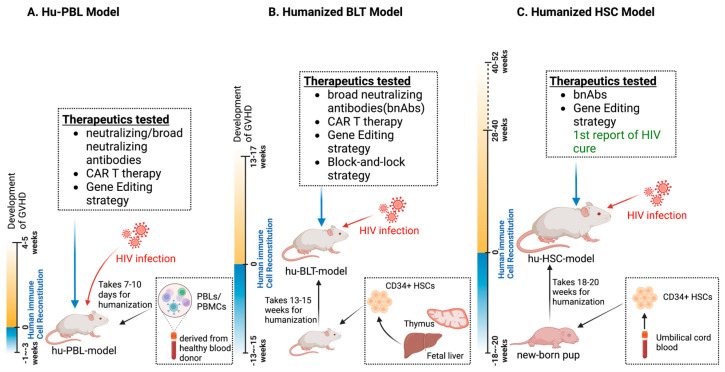
Humanized mouse models for preclinical HIV therapeutics. (**A**) The hu-PBL model employs peripheral blood mononuclear cells (PBMC/PBLs) from human donors that are injected into immunocompromised mice. This model typically reaches functional maturity within 1–2 weeks but is highly susceptible to graft-versus-host disease (GVHD) within 4–5 weeks of cell implantation. The hu-PBL model is commonly used for drug discovery and quick screening of antiretroviral drug combinations as well as novel antiretroviral therapies. (**B**) The hu-BLT model involves the transplantation of human HSCs and fetal liver and thymus tissues into immunocompromised mice to generate a more complete human immune system. This model requires 13–15 weeks for human immune cells to reach full functional maturity, and it has been extensively used as a chronic HIV infection model for HIV latency and cure studies, enabling therapy testing for more than 10 weeks. (**C**) The hu-HSC model utilizes umbilical-cord-blood-isolated CD34+ hematopoietic stem cells (HSCs) that are transplanted into newborn pups to generate functional human immune systems. It necessitates 18–20 weeks for full maturation and allows for long-term testing of HIV reservoir establishment and therapeutic targeting studies for up to one year. The first report of an HIV cure in a humanized mouse model used sequential long-acting ART and CRISPR-based gene editing treatments. This model has better clinical translational potential than the other models.

In spite of the advantages of rodent models, a few limitations do exist, including the shorter lifespan of human cell persistence in vivo, which limits long-term efficacy studies. Most of the humanized mouse models used for HIV latency and cure studies are developed on either well-characterized NSG, NOG-SCID, or BRG genetic backgrounds [128,129,130,131,132]. Depending on the different humanization methods, the three main mouse models are deployed to assess either short-term or long-term HIV persistence, virus–host interactions, and therapeutic developmental investigations that serve to best target the viral reservoirs (Figure 1). 

### 5.1. Hu-PBLs

To better understand the progression of human disease and to allow for the testing of newly developed therapeutics in vivo in a reasonable, shorter time duration and in a smaller animal model with the presence of the required human cells, a new model was developed by researchers. This model used the injection of human peripheral blood leukocytes (PBLs) into a severely immunodeficient mouse system and is popularly called the hu-PBL mouse model. It has a long history as a platform for not only studying HIV infection but also for the quick screening and testing of newly developed therapeutic approaches [128]. This model is regarded as one of the rapid and direct means of developing short-term humanized mice via the intraperitoneal injection of human peripheral blood mononuclear cells (PBMCs) or PBLs for experimental testing within 1–3 weeks [128]. The major cell types required for HIV-infection studies, such as human T cells, myeloid cells, and B cells (albeit at low level), are well reconstituted in PBL mice in various compartments including the lung, liver, spleen, and brain upon cell transplantation; however, these mice lack multilineage hematopoiesis and primary immune responses [133]. Due to the ease and convenience of generating PBL mice, this model has been extensively used as a rapid screening model system to test newly developed antiretroviral regimens for HIV studies [134,135,136]. Antiretroviral drugs can only suppress viral replication but cannot eliminate HIV because of HIV latency, so the hu-PBL model has been deployed in other approaches to study HIV elimination which include bnAbs, chimeric antigen receptor (CAR) T therapy, and gene-editing technologies alone or in combination with ART regimens to better understand the efficacies of these new therapies, as reviewed in [137]. Initial studies using either a single antibody or a cocktail of three neutralizing antibodies over a 15-day treatment period failed to show an inhibitory effect on the viral loads in established HIV-1 infections with minimal success in hu-PBL models, with the detection of neutralization escape variants within 5 days [138]. The authors posit that the failure to achieve antiviral efficacy using neutralizing antibodies was mainly due to the of lack of restricted neutralizing antibody effector functions like complement activation and antibody-dependent cellular cytotoxicity (ADCC) in the hu-PBL mouse model, causing the researchers to use more improved humanized mouse models with better functional human immune systems for future efficacy studies. 

CAR T cell therapy has shown success in the treatment of cancer and as such has recently brought hope to the field of HIV research. CAR T cells can directly act against HIV infected cells, thus providing an alternative HIV cure strategy [139]. Recent testing of CAR T cells in the laboratory and later in hu-PBL mice has shown promise for the clearance of viral infection [140]. The authors first injected CD8-depleted blood mononuclear cells into NSG mice, followed by an injection of HIV-1 infected CD4+ T cells at day 12 after lymphocyte transplantation. After infection, the mice were treated with daily ART for 4 days and subsequently infused with CAR T cells for 40 days post PBMC transplantation, which was equivalent to 28 days post infection. The authors found that the infusion of HIV-specific CAR T cells into hu-PBL mice led to undetectable plasma HIV RNA 10 days after ART removal, and the mice continued to show lower levels of viral load (compared to the control) until the end point. Combined with other evidence from this study, HIV-specific CAR T cells demonstrated efficacy in promoting T cell survival and protecting CD4+ T cells from HIV-1-mediated depletion, focusing on attenuating viral rebound after ART cessation [140]. Later, in another study, an advanced form of CAR T cells targeting multiple sites of the HIV-1 envelope was developed and tested in an acute infection model which showed a reduction in viral replication. The study used an intrasplenic injection of 10 million infected lymphocytes and 1 million CAR T cells into hu-spl-NSG mice. At the endpoint, which was seven days after infection, the spleens were harvested and analyzed for three populations: CD4+, CD8+, and CAR T cells. The results demonstrated that the CAR-T cells eliminated > 97% of HIV-1 infection compared to the control group due to the CAR-mediated elimination of HIV-infected lymphocytes, suggesting a potential CAR T cure approach [141]. However, the data obtained were short-term observations and may not reflect true latency. The long-term HIV-protection effects of these modified CAR T cells must be further examined using suitable long-term model systems prior to clinical translation.

Yet another approach used by researchers targeting HIV persistence in model systems is gene editing. Over the past decade, engineered nucleases were found to be capable of recognizing target DNA sequences. This was followed by viral cleavage in attempts to diminish or eliminate HIV-1 infection. These endonucleases include zinc finger nucleases (ZFNs), transcription-activator-like effector nucleases (TALENs), and, more recently, CRISPR-associated nuclease 9 (Cas9). These are collectively known as anti-HIV gene therapies. The approaches are designed to recognize specific regions of the HIV genome and excise the HIV proviral DNA or inactivate the host genes responsible for affecting parts of the viral life cycle, including those that affect viral entry into cells and active replication [142,143,144,145]. The next round of studies was performed in hu-PBL mice to test the efficacies of ZFNs and TALEN-based gene editing. The data showed that engrafted CD4+ T cells were only modestly protected against HIV-1 infection. Due to detectable off-target effects and the lower gene-editing efficacies of ZFNs and TALENs in hu-PBL mice [146,147,148], the emergence of the CRISPR system took HIV elimination research to a whole new level [149]. One study demonstrated that the engraftment of CRISPR-Cas9-edited CCR5, performed in human T cells in hu-PBL mice, caused resistance to HIV-1 infection. Notably, the CRISPR-edited T cell population survived 45 days after infection compared to the non-edited cells [150]. This hu-PBL study emphasized the critical role of CRISPR-edited CCR5 T cells in HIV infection and reservoir establishment and introduced a new preventive approach to reducing viral reservoirs [150]. However, the mouse model used in this study had a less reflective immune cell population to mimic human disease; the interplay and segregation of CRISPR-edited T cells with other important immune cells such as B cells, NK cells, DCs, and macrophages have not been developed to date. Thus, the long-term maintenance and efficacy of CRISPR-edited T cells in a more advanced animal model system with a more robust transplanted human immune system is needed. When all these aforementioned factors are present, the results obtained may better represent “true” HIV latency. 

While the use of hu-PBL mice can enable short-term HIV-1 therapeutic studies involving human T cells, the model has limitations in that it commonly develops xenogeneic graft-verse-host-disease (GVHD). GVHD occurs within three to five weeks following human cell implantation [151]. For this reason, the timeline necessitates that the establishment of HIV-1 reservoir and conducting the targeting experiments must occur within 5 weeks from the day of cell implantation in this animal model system, which is not an ideal model of HIV-1 latency [152]. The induction of uncontrolled B and T cell activation makes the hu-PBL mice inappropriate for long-term HIV studies [133].

### 5.2. Hu- Bone Marrow/Liver/Thymus (BLT) Mice

To overcome the limitations of hu-PBL model, the human BLT model, popularly known as “BLT humanized mouse model”, was developed and it is widely used to study HIV persistence and cure strategies. The generation of the BLT model involves multiple procedures, beginning with whole-body irradiation with a sublethal dose, followed by the transplantation of human fetal liver tissue under the kidney capsule of the mouse and a separate procedure in which the thymus tissues are transplanted under the renal capsule within three days post irradiation. One to two weeks after the implantation of human fetal liver and thymus tissues, the mice are injected with CD34+ hematopoietic stem cells (HSCs) isolated from the autologous fetal liver to generate the human immune system [131,153], and this model has a long-term functional human immune system compared to the hu-PBL model. Compared to hu-PBL mice, multilineage hematopoiesis, a primary immune response, and the education of T cells are the primary advantages of BLT mice, which give rise to a more stable population of mature T cells, as well as other immune cells including B cells, NK cells, dendritic cells, and monocytes/macrophages. With the advantages the model possesses, BLT mice not only support chronic HIV infection studies but also allow for the establishment of long-term latency for more than two months post infection and serve as an attractive model for studying HIV reservoirs and testing HIV eradication strategies in vivo [154]. The successful use of a latency-reversal agent, SUW133, in hu-BLT mice was demonstrated. Hu-BLT mice were first infected with HIV for six weeks and then treated with ART for 4.7 weeks. SUW133 and two rounds of natural killer (NK) cell treatments were administered sequentially before ART was interrupted. Plasma viral levels and tissue DN-/RNA levels were evaluated after ART cessation. Interestingly, among the ten mice in the LRA+ NK group, four displayed no viral rebound, whereas the mice injected with either LRA or NK cells exhibited noteworthy delays in viral rebound when compared to the control group [155]. No HIV DNA was detected in the tissues of the dual-treated mice, whereas the control group had HIV DNA in their lymph nodes and bone marrow, indicating the important role of LRAs accompanied with other innate immune regulators in the hu-BLT model in the quest for an HIV cure [155].

After several unsuccessful initial trials utilizing traditional neutralizing antibodies for HIV suppression in humanized mouse models, the recently identified potent bnAbs were found to be highly efficacious in targeting a versatile virus population [156]. This discovery prompted the testing of these potent bnAbs in vivo. First, bnAbs were isolated from patients via single-cell antibody cloning techniques and then tested for their efficacy in HIV-infected hu-BLT mice [157]. The HIV-specific bNAb PGT121 was evaluated in both immunocompromised C57BL/6 and hu-BLT mice. The study found that the administration of PGT121 in hu-BLT mice resulted in a significant reduction in viral load. The BLT-mice were first infected and then treated with PGT121 at 17 days post infection. The plasma viral load and serum PGT121 concentration were monitored until 21 days post treatment. Serum concentrations of PGT121 were maintained in hu-BLT mice for 7 weeks [158]. These results demonstrate the translational value of bnAbs as therapeutic treatments and their potential for targeting viral reservoirs without ART. However, since hu-BLT mice have incomplete adaptive immunity, few antibodies are generated against drugs (PGT121) (anti-drug antibodies). This loss of specific antibody formulation during the treatment may preclude its appropriate translation to humans. The half-life of PGT121 of over 40 days in hu-BLT mice did not recapitulate PGT121 immune responses as would be observed in humans [159]. Thus, an additional evaluation of the clearance of antibodies in other models is required before this method can be translated successfully to the clinic. 

To achieve long-term viral suppression, a long-term functional effector T cell population that can recognize appropriate antigens and kill the infected cells needs to be realized, which is the basic mechanism of the CAR T cell approach. While most of the in vitro or ex vivo studies have shown promising antiviral effects of CAR T cells [160,161,162], a few groups have examined the prolonged effects of CAR T cells in vivo [163,164]. One study provided the first proof-of-concept long-term establishment of effector T cells in hu-BLT mice [163]. Without any HIV-1 infection, the authors first developed a genetically modified HSC-based therapy and then characterized the effector T cell population and the responses against the stimulating antigen. A large, mature population of HIV-specific CD8+ cells were identified in these mice 7 weeks post transplantation. The cells generated IFN-γ in response to HIV-antigen stimulation, indicating their ability to recognize and kill viral-antigen-presenting cells. However, this cell population was not maintained for a long period of time, which was interpreted as the lack of a correct hematopoietic niche for the survival and growth of the implanted genetically modified HSCs in the hu-BLT mice [164]. Zhen et al transplanted hu-BLT mice with triple-CAR-modified HSCs and then challenged the mice with HIV infection for 5 weeks. Mice with higher percentages of CAR cell expression exhibited higher degrees of viral suppression (decreased plasma RNA and blood DNA) and restored CD4/CD8 immune profiles, whereas the hu-BLT mice with lower CAR expression levels showed limited protection against HIV infection [164]. These results indicate that although the protection offered by CAR-expressing cells against HIV-infection appears promising, their maturation and stability in vivo is still a major concern for translation and requires further investigation. 

In addition to CAR T cell therapy, the hu-BLT model has been employed for testing anti-HIV gene-editing strategies, particularly the recently discovered potent CRISPR-Cas9 system. One study examined the AAV-mediated delivery and excision efficacy of the CRISPR system in both acute and chronic HIV infection mouse models and provided a comprehensive understanding of how CRISPR works in an in vivo system [149]. The first studies tested CRISPR in HIV-infected Tg26 mice. Multiple doses of the AAV-DJ/8-mediated quadruplex sgRNAs/Cas9 system were used to target sequences in the gag and LTR regions of the HIV genome in the infected mice. After 20 days of CRISPR treatment, successful proviral DNA excision was observed in major organs such as the brain, kidney, liver, and lung. The efficacy of the system was further tested in a hu-BLT mouse model supporting chronic HIV infection. Two BLT mice were inoculated with HIV_NL-BaL_-eLuc reporter virus and then administered a single injection of the quadruplex sgRNAs/Cas9 system. Four weeks after the AAV-DJ/8 injection, the mice were euthanized and genotyped utilizing specific primers to evaluate viral excision. The results indicated that HIV cleavage was efficiently induced in the spleen, lungs, heart, colon, and brain with minimal off-target effects, suggesting the efficient gene delivery of these AAV viral vectors and the high efficacy of the quadruplex CRISPR-based gene editing system [149]. The study provided valuable information on the expression duration of the AAV vector in vivo and the potential of the CRISPR-mediated excision of HIV in a humanized mouse model. The mice were not maintained for a longer time for further investigation, due in part to the lack of other viral suppressive interventions. 

The BLT model has also been employed in testing a new cure approach named “block-and-lock”, which aims to achieve and maintain permanent long-term silencing of HIV expression, preventing the virus from rebounding after ART withdrawal. Early attempts using didehydro-Cortistatin A (dCA), a Tat competitive inhibitor, aided in the establishment of an “inactivation” state of the provirus in which no virus production was observed until 22 days after ART withdrawal [165]. The effect of dCA on HIV latency was further tested in the hu-BLT humanized mouse model [166]. Prior works were performed on infected BLT mice challenged with HIV-1JRCSF followed by ART to suppress viral replication and record functional outcomes [166]. All treatments were halted nine weeks following viral infection. Plasma viral loads were measured every 3 or 4 days until viral rebound. It was found that four weeks of dCA co-administration with ART significantly delayed viral rebound, with lower viral levels upon treatment interruption for 16 days compared to ART [166]. This preliminary work suggested that the block-and-lock approach could delay viral rebound; however, it failed to have a long-term “locking” effect upon ART withdrawal. Other “block and lock” candidates, such as epigenetic suppression small molecules, have shown short-term transcription “blocking” with limited toxicity [167,168,169]. However, despite some promising results, their development is still in its early stage. It remains to be seen whether and how these molecules will facilitate long-term suppression of the provirus, particularly in hu-BLT mice with longer-term stable human lymphocyte reconstitutions. Therefore, further investigations are required to understand the results of these candidates as curative HIV approaches [166,170]. 

While hu-BLT mice have several advantages over Hu-PBL mice, especially permitting the long-term systemic human immune system reconstitution, as summarized in Figure 1B, the major drawback of the BLT mouse model lies in the development of GVHD-like symptoms, particularly alopecia, blepharitis, and liver inflammation, 3–4 months after study initiation. This renders the model unsuitable for studying long-term HIV latency and reservoir targeting [121,171]. BLT mice also present higher frequencies of immature B cells and lower frequencies of NK cells compared to the human immune profile. Additionally, the reconstitution of monocytes/macrophages in this model is incomplete as the majority of human monocyte/macrophages were in detected in the lymph nodes and spleen [170,172,173,174,175]. Recent restrictions imposed since 2019 by federal laws in developed countries, including the USA, on obtaining and using fetal liver cells for research is another limitation for the generation and testing of therapeutic paradigms in hu-BLT mice, rendering the need for an alternative small model system [176]. 

### 5.3. Hu-HSC Model

Because of the complexity and restricted sources in generating hu-BLT mice, another mouse model, the hu-HSC mouse, which can also support the long-term systematic engraftment of a functional human immune system and is easier to generate, is being widely used in HIV-1 research. The hu-HSC model is generated via either the intravenous or intrahepatic injection of purified human CD34+ hematopoietic stem cells into irradiated newborn mice with immunodeficient genetic backgrounds [129,177,178,179]. HSCs can be derived from a variety of sources; most researchers isolate them from umbilical cord blood or aborted fetal livers. The hu-HSC model shares many similarities with the hu-BLT model; both models support multilineage hematopoiesis and have primary immune responses; however, the T cells in the hu-HSC model are not HLA restricted due to the absence of a human thymus [178]. In the hu-HSC model, the human T cells, which are the major targets of HIV infection, mature in a mouse thymus compared to the hu-BLT model in which the T cells are educated in an implanted human origin thymus capsule. Despite the use of non-HLA-restricted T cells, successful, long-lasting HIV infections have been achieved in hu-HSC mice via multiple routes [180,181,182]; importantly, key features of HIV latency establishment were replicated in these mice after ART treatment [183,184]. Moreover, since it is convenient to obtain HSCs from cord blood from different hospital settings with prior approvals compared to obtaining fetal tissues, and because it is easier to perform the cell isolation, purification, characterization, and transplantation procedures, the hu-HSC model has increasingly advanced our understanding of HIV latency and therapeutic intervention studies. 

Several initial studies on the HIV cure approach have utilized the hu-HSC model to test the efficacy of bnAbs during HIV infection. In one study, researchers first inoculated hu-HSC mice with HIV-1_NL4-3_ and subsequentially administered five different bnAbs, either alone or in combination [185]. While the single-antibody treatment initially reduced the viral loads when provided 20 days post infection, viremia quickly rebounded to pretreatment levels within two weeks with detectable, HIV-resistant mutant strains to each bnAb tested in these mice [185]. Importantly, most of the mice treated with a combination of either three or five bnAbs exhibited sustained viral control for a prolonged period (20 days post infection) until the clearance of the bnAbs from the system, which varied between individual mice. Eventually, viral rebound was observed in all animals, ranging from 20 to 60 days post treatment, with the longest delay being 100 days. Notably, no evidence of bnAb-resistant mutants was detected from these triple or penta-mix bnAbs-treated mice [185], suggesting the translational potential of bnAb combinations for future HIV elimination studies. Another group treated the infected hu-HSC mice with a combination of three different bnAbs, along with ART treatment. ART was first administered 3 weeks post infection and was maintained for 3 weeks. Combinatorial bnAbs were administered shortly after initiating ART treatment for another four weeks before ART interruption. The viruses remained sensitive to neutralization in all the animals, and the viral loads were similar to the pretreatment levels in rebounded animals when serum antibody titers were low or undetectable. Viral rebound was reported in most animals approximately 8–12 weeks after ART cessation [186]. Consistent with this finding, another group treated hu-HSC mice with ART with or without bnAbs. Following HIV infection, significant recorded delays in viral rebound were observed in hu-HSC mice treated with a combination of bnAbs and ART for 74–107 days when compared to the mice treated with ART alone (28–84 days), indicating that early bnAb intervention (4 days post infection) was able to delay or suppress the establishment of the viral reservoir [187]. Altogether, utilizing bnAbs alone or in combination with ART not only suppressed active HIV replication but also delayed viral rebound and may have had an effect in reducing the viral reservoir over the course of chronic infection. However, these studies also suggest that even with the use of a multiplexed bnAb intervention, the reservoirs were established at the time of ART suppression or prior to ART treatment, highlighting the complexity and challenges remaining to be addressed to determine the potential for any complete eradication of HIV. 

To date, only two studies have achieved a sterilizing cure for HIV, and both were reported in hu-HSC mice [188,189]. In 2019, Dash et al. reported the first complete HIV elimination from all the body compartments analyzed from a subset of infected human-HSC-reconstituted mice via the sequential administration of nanoformulated, long-acting ART treatment and AAV9-mediated CRISPR targeting HIV-LTR-Gag. In this study, Hu-HSC mice were first infected with HIV-1 for 2 weeks, followed by treatment with a combination of four (DTG, 3TC, ABC, and RPV) nanoformulated, long-acting antiretroviral drugs for four weeks to achieve active viral suppression. ART was then discontinued, and a single dose of CRISPR-Cas9 targeting HIV-LTR-Gag was administered. The mice were then followed for 2 months to monitor viral rebound. Eight out of the 23 mice that received sequential dual treatment showed no viral rebound 8 weeks after ART interruption, while all the mice in the single-treatment and untreated groups showed rebound viremia. Moreover, no viral RNA or DNA, analyzed using multiple highly sensitive assays with detection limits of 1–2 viral copies that included nested qPCR, digital droplet PCR, RNAscope, and viral outgrowth assays, were detected in the tissue compartments of the aviremic mice. No off-target effects were detected in the CRISPR-treated animals, indicating the potential translational value of the CRISPR treatment in the quest for an HIV cure. Importantly, the presence of replication-competent virus in various tissues in the single-treatment groups (CRISPR or ART alone) demonstrated that this mouse model is a long-term reliable model for studies of HIV latency and cure [188]. In 2023, we achieved increases in HIV elimination rates of up to 58% using a sequential long-acting ART and dual CRISPR-editing strategy targeting host-CCR5 and HIV-LTR-Gag in a Hu-HSC infection model, suggesting that the model is an ideal and valuable model for testing novel HIV cure approaches [189].

This hu-HSC model has recently been employed to recapitulate replication-competent viruses from virally suppressed peripheral, myeloid, and CNS compartments [190]. The viral outgrowth assays published so far require 50–100 million cells for adoptive engraftment for the validation of an HIV cure, which are not always possible to obtain from patients. The newly reported in vivo viral outgrowth assay needs only 1–2 million cells from virally suppressed animals, and the Hu-HSC recipient mice were able to recapitulate and spread the viral infection to all body compartments, including the brain [191], highlighting the importance of this model, as summarized in Figure 1C, and for future strategies targeting latent reservoirs for HIV cure validations.

## 6. Perspectives

Given the successful application of hu-HSC mice in long-term HIV studies due to the presence of a functional human immune system that can last up to one year (recent observations), this model is considered by far the closest small animal translational model system for HIV latency and cure studies. However, limitations do exist. First, the human T cells are produced and educated in the mouse thymus, which is of some concern for their development status [123,178]. A recent study, however, revealed that human T cells were preferentially developed in NRG-hu-BLT mice with more predominant and well-educated T cell populations than NRG-hu-HSC mice [183]. The same study also suggested that there are no significant differences in responses between hu-BLT and hu-HSC mice when used to study HIV-1 replication, pathogenesis, and therapeutics [183]. The primary advantages of hu-BLT mice over hu-HSC mice are in studies involving human HLA class I and class II restricted T cell responses. The second limitation is the underdeveloped lymph nodes and lower level of human cell reconstitution in the gut and mucosal tissues of the hu-HSC mouse. However, no significant differences were observed in HIV long-term studies compared to other animal model systems. Third, hu-HSC mice harbor fewer cells of human origin (microglia, macrophages, and astrocytes) responsible for carrying latent virus in CNS compartments [192,193]. Encouraging recent studies have described the development and successful use of another hu-HSC model with human microglia [194]. Human interleukin-34, a key macrophage–microglial differentiation factor, was introduced into the NOG strain, which led to the successful reconstitution of human microglia-like cells in the mouse brain, thus allowing for the study of principal CNS viral reservoirs. Additionally, this model was able to recapitulate neuroHIV. This was evidenced via transcriptomic analyses of antiviral, inflammatory, and human-specific molecular signatures, which offer insights into viral persistence in the brain. 

**Figure 2:**
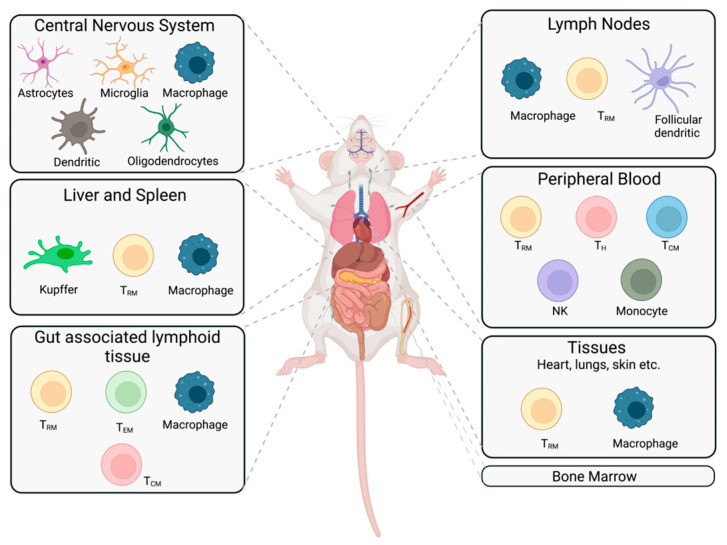
HIV reservoir target cells in a suitable small animal model system to study HIV latency. Cells demonstrated to harbor latent HIV in different tissues are shown. We propose an ideal humanized rodent model system that depicts HIV reservoirs spread across various tissue types around the body. The majority of latent virus is found in CD4+ resting memory T cells (T_RM_), along with other CD4+ T cell subsets (T_EM_, T_CM_, _and_ T_H_). MP reservoirs are in a range of tissue compartments. In addition to having a peripheral functional human immune system, the ideal humanized model system should have abundant cells of human origin which include T cells, B cells, dendritic cells, monocytes, and macrophages that can be maintained for a year for long-term HIV latency studies and subsequent targeting of those reservoirs for ultimate HIV elimination and for clinical translation. T_EM_ = T effector memory; T_CM_ = T central memory; T_H_ = T helper; NK = natural killer.

CD34 mouse models are used in a variety of platforms and are employed as mainstream small animal models to study HIV pathobiology, drug safety and efficacy, latency, and reservoir targeting and in studies that can modulate the immune system. The optimization of preclinical small animal models is the key to testing the up-to-date clinical advances in HIV therapeutic developments, which can ensure the improved translation of various preclinical results into interventions and can ultimately benefit patients. In summary the hu-HSC background represents an ideal long-term model for studying HIV-1 latency and cure, with diverse cell types reconstituted in immunologically active compartments and well-defined human cells that can last for more than six months to one year, as described in Figure 2. Its advantages include the simplicity of the mouse generation procedure, abundant sources of CD34+ HSCs from umbilical cord blood, and the greatly reduced incidence of GVHD (less than 5%), making it the leading mouse model for studying and validating HIV cure strategies prior to assessments in NHP models or clinical translation.

## Data Availability

Not applicable.

## Abbreviations

MPs	Mononuclear phagocytes
HIV	Human immunodeficiency virus
ART	Antiretroviral therapy
CNS	Central nervous system
CTL	Cytotoxic T lymphocyte
MOI	Multiplicity of infection
PKC	Protein kinase C
PLWH	People living with HIV
MDMs	Monocyte-derived macrophages
NHP	Nonhuman primate
BnAbs	Broadly neutralizing antibodies
CAR T	Chimeric antigen receptor T cell
SCID	Severe combined immunodeficiency
GVHD	Graft-versus-host disease
HSC	Hematopoietic stem cells
PBLs	Peripheral blood leukocytes
PBMCs	Peripheral blood mononuclear cells
ADCC	Antibody-dependent cellular cytotoxicity
ZFNs	Zinc finger nucleases
TALENs	Transcription activator-like effector nucleases
CRISPR-Cas9	Clustered regularly interspaced short palindromic repeats and CRISPR-associated protein 9
NK cell	Natural killer cell
HAND	HIV associated neurocognitive disorders
SIV	Simian immunodeficiency virus
SHIV	Simian human immunodeficiency virus
AAV	Adeno-associated virus
LTR	Long terminal repeat
